# KniMet: a pipeline for the processing of chromatography–mass spectrometry metabolomics data

**DOI:** 10.1007/s11306-018-1349-5

**Published:** 2018-03-16

**Authors:** Sonia Liggi, Christine Hinz, Zoe Hall, Maria Laura Santoru, Simone Poddighe, John Fjeldsted, Luigi Atzori, Julian L. Griffin

**Affiliations:** 1 Department of Biochemistry and Cambridge Systems Biology Centre, University of Cambridge, Cambridge, UK; 2 Section of Pathology, Department of Biomedical Science, University of Cagliari, Cagliari, Italy; 3 Agilent Technologies, Santa Clara, CA USA

**Keywords:** Metabolomics, Data processing, GC–MS, LC–MS

## Abstract

**Introduction:**

Data processing is one of the biggest problems in metabolomics, given the high number of samples analyzed and the need of multiple software packages for each step of the processing workflow.

**Objectives:**

Merge in the same platform the steps required for metabolomics data processing.

**Methods:**

KniMet is a workflow for the processing of mass spectrometry-metabolomics data based on the KNIME Analytics platform.

**Results:**

The approach includes key steps to follow in metabolomics data processing: feature filtering, missing value imputation, normalization, batch correction and annotation.

**Conclusion:**

KniMet provides the user with a local, modular and customizable workflow for the processing of both GC–MS and LC–MS open profiling data.

## Introduction

Among the several analytical techniques employed within metabolomics, gas and liquid chromatography coupled with mass spectrometry (GC– and LC–MS) are the most commonly used in metabolomics studies as they allow the identification of a large number of diverse molecular species. However, the plethora of samples analyzed during high-throughput screenings, the number of processing steps, and the required computational competences and resources often represent a bottleneck that renders these analyses slow and potentially inaccurate. Hence, utilization of standardized procedures is fundamental for reliable and reproducible results (Meier et al. 2017; Rocca-Serra et al. 2016; Sandve et al. 2013). Several protocols have been proposed or are currently being developed (Beisken et al. 2014; Di Guida et al. 2016; Dunn et al. 2011a; Giacomoni et al. 2015; Guitton et al. 2017; Rocca-Serra 2017; Southam et al. 2017; Weber et al. 2017). However, they are not free from pitfalls, the main ones being related to a high level of computational expertise needed for their local installation, utilization and implementation. The alternative provided by web-based services can be affected by inadequate stability, security and performance in handling a large number of samples, or sensitive data.

For these reasons, the KNIME Analytics Platform (Berthold et al. 2007) was used to build a vendor-independent processing workflow. KniMet (Liggi 2017) joins several steps required to process GC– and LC–MS metabolomics data, outputting a data matrix normalized, annotated and filtered from inconsistently detected features in a semi-automated, documented and reproducible analysis.

## KniMet features

**Fig. 1:**
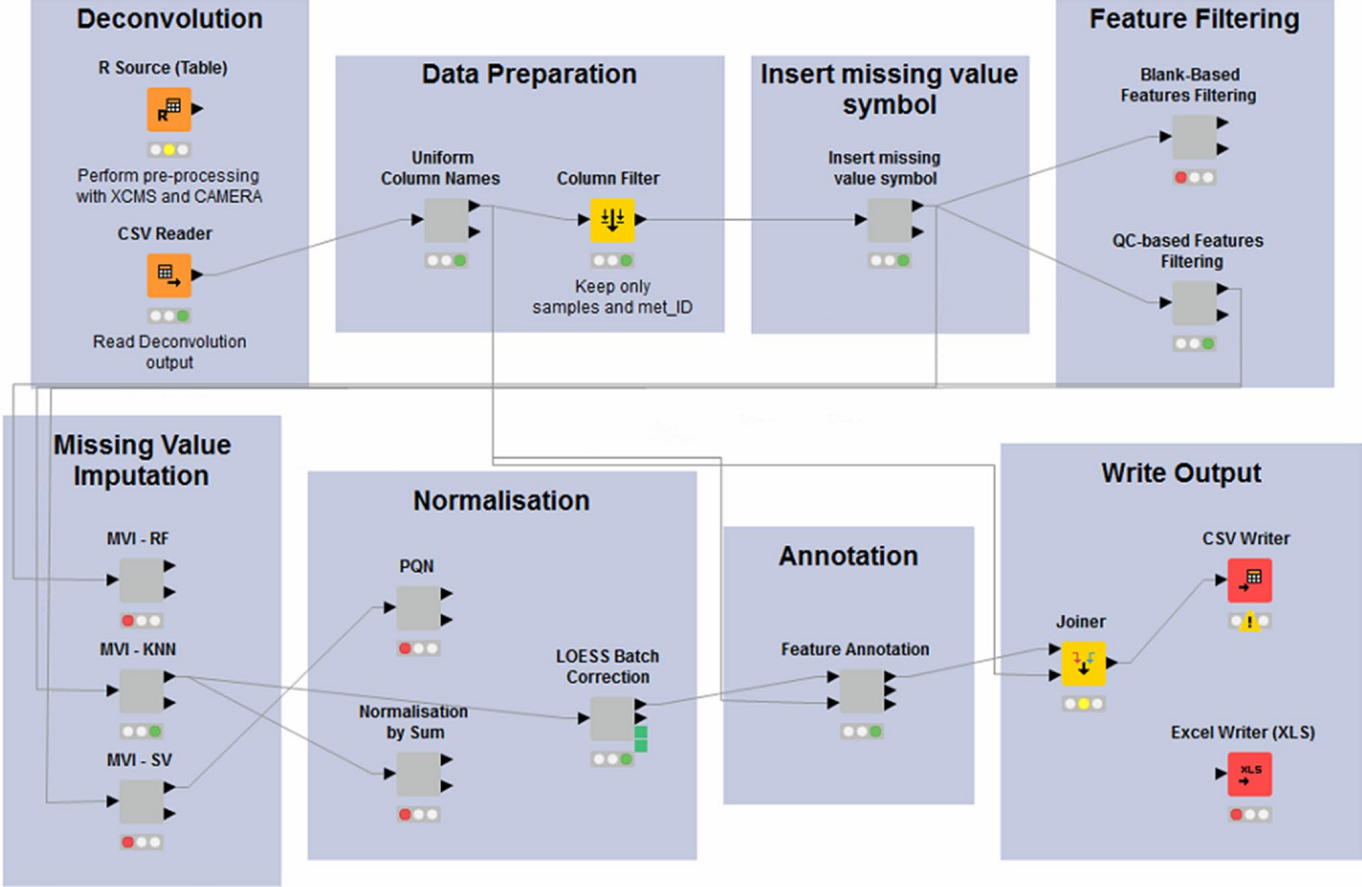
The KniMet pipeline comprises different steps for the post-processing of metabolomics data each one enclosed in a square in this representation. Most of these steps can be performed with multiple tools, allowing the user to combine them in the most appropriate way for the specific dataset studied

The steps performed by KniMet comprise data deconvolution, feature filtering, missing value imputation, normalization and features annotation. For each one of these steps there are several options, as shown in Fig. 1 and described below, allowing users to utilize the most appropriate tool for the specific case study at hand.

### Data deconvolution

GC– and LC–MS data in mzXML or CDF format (previously converted with, for instance, *Proteowizard* [14]) can be deconvoluted internally with the R (R Core Team 2014) library *XCMS* (Smith et al. 2006), or by integrating into KniMet the OpenMS nodes (Pfeuffer et al. 2017). Alternatively, this step can be performed externally with either the locally installed R instance, XCMS online [17] or a vendor software. In this case, the obtained data matrix can then be imported in the pipeline and subjected to further analysis. For instance, a dataset obtained using the Agilent 6560 Ion Mobility Q-TOF LC–MS was deconvoluted with MassProfiler from the MassHunter Workstation Software suite (Agilent Technologies, Santa Clara, USA), fed into KniMet and then subsequently processed using downstream tools.

### Feature filtering

Periodic injections of pooled samples, also known as quality controls (QCs) are used to account and correct for analytical variation, based on the assumption that QCs should contain all the signals present in the samples. Hence, if the instrument performance is stable, these signals should be consistently detected across the run, while only unstable metabolites or contaminants would be detected inconsistently (Dunn et al. 2011a). According to these principles, all features whose signal is missing in more than a given percentage of QCs (defined by the user, default 50%) and whose Relative Standard Deviation across the QCs is higher than a threshold (set by the user, default 20%) are deleted. An alternative method not based on pooled samples was implemented to account for experimental setups in which QCs are missing and/or the user would rather perform feature filtering based on other samples, such as blanks. In this case, only features whose average intensity in the samples is higher than their average intensity in blanks multiplied by a user-defined factor are retained. Moreover, features are filtered if they are missing in more than a user-defined percentage of samples.

### Missing values handling

Missing values in the data matrix can occur for several reasons, such as (i) missingness of a feature in one (class of) sample(s) and not in another, (ii) concentration of a metabolite in a sample lower than the analytical limit of detection (iii), or inaccurate pre-processing with lack of deconvolution of a feature. An appropriate evaluation of the reasons behind the presence of missing values in the data matrix, and their consecutive imputation, is fundamental to avoid biased statistical results (Di Guida et al. 2016; Gromski et al. 2014). In this application, missing values imputation can be performed with either Random Forest (RF) or K-Nearest Neighbour (KNN) algorithms, implemented as R scripts using the libraries *missForest* (Stekhoven and Buhlmann 2012) and *impute* (Hastie et al. 2016) respectively, or Small Value replacement (SV), i.e. half of the minimum value found for a given feature in given sample.

### Normalization

Among the several normalisation methods available, Probabilistic Quotient Normalization (PQN) (Dieterle et al. 2006) and Sum Normalisation have been implemented in KniMet as they are the most commonly used in MS-based metabolomics data (Di Guida et al. 2016). PQN consists of: (i) calculation of a reference spectrum (or vector) as the median of each signal in the entire set of samples or, if available, in the QCs; (ii) division of each signal found in the samples by the value for the same signal in the reference spectrum to obtain a list of quotients; (iii) division of the original data matrix for the median of these quotients. On the other hand, in Sum normalization each feature in a given sample is divided by the sum of all features in that sample and multiplied by 100.

Peak drift is an issue in metabolomics data obtained from LC–MS instruments, as a number of factors which vary with time can affect the results. In the case of batch-effects being present, batch-correction normalization can be performed to merge samples measured in different analytical blocks. Among the several methods available, the robust locally estimated scatterplot smoothing (LOESS) signal correction (RLSC) method based either on QCs or all samples (Dunn et al. 2011b; Thévenot et al. 2015) were implemented utilizing the R scripts developed by the Workflow4metabolomics team (Giacomoni et al. 2015).

### Metabolite annotation

Metabolite annotation based on accurate mass match with the Human Metabolome Database (Wishart et al. 2017) and the LIPID MAPS database (Fahy et al. 2007; Sud et al. 2006) was implemented by integrating the *AccurateMassSearch* functionality of OpenMS.

## Conclusions

KniMet is a KNIME-based pipeline for the analysis of metabolomics MS data. This platform is easy to install and run locally, providing the user with full control of the analysis. Indeed, the modular structure of the platform allows the pipeline to be modified based on the nature of the data to be processed, and hence be applied to datasets derived from different analytical and/or experimental setups. The resulting tables containing all the analyzed samples and the detected metabolic features can be exported and are ready for further statistical analysis. A recent and published example of its application is the processing of both GC– and LC–MS data of fecal samples from patients affected by Inflammatory Bowel Diseases compared with a population of healthy subjects, with the aim to identify new biomarkers for the disease (Santoru et al. 2017).

Moreover, KniMet is fast and does not require particularly high computational power: the post-processing of the R data package faahKO (Saghatelian et al. 2004) as described in the user guide, takes less than 10 s and a peak of 1331.65 MB of memory consumption on a PC with Intel® Core™ i7.

In conclusion, with the KniMet application we provide the user with a highly flexible, fully customizable and user-friendly platform which includes the key processing steps of metabolomics data.

## Availability and implementation

KniMet is freely available under the 3-Clause BSD License at https://github.com/sonial/KniMet along with usage instructions and example data.
